# AXL Inactivation Inhibits Mesothelioma Growth and Migration via Regulation of p53 Expression

**DOI:** 10.3390/cancers12102757

**Published:** 2020-09-25

**Authors:** Wei Song, Hao Wang, Minmin Lu, Xinxin Ni, Nacef Bahri, Shuihao Zhu, Limin Chen, Yuehong Wu, Jieqiong Qiu, Jonathan A. Fletcher, Wen-Bin Ou

**Affiliations:** 1 Zhejiang Provincial Key Laboratory of Silkworm Bioreactor and Biomedicine, College of Life Sciences and Medicine, Zhejiang Sci-Tech University, Hangzhou 310018, China; song786722358@163.com (W.S.); wanghao9611@126.com (H.W.); 15067150649@163.com (M.L.); nixin3877@163.com (X.N.); zsh19950701@163.com (S.Z.); clm2285183092@163.com (L.C.); wuyh77@zstu.edu.cn (Y.W.); qiujieqiong@zstu.edu.cn (J.Q.); 2 Department of Pathology, Brigham and Women’s Hospital and Harvard Medical School, Boston, MA 02115, USA; nbahri@bu.edu (N.B.); jfletcher@bwh.harvard.edu (J.A.F.)

**Keywords:** mesothelioma, AXL, p53, regulatory loop, target therapies

## Abstract

**Simple Summary:**

Previous studies have shown that AXL is a crucial protein that is activated in mesothelioma to cause growth and invasiveness. p53 is the tumor suppressor protein that is most frequently inactivated by mutations in human cancers, but these p53 inactivating mutations are not common in mesothelioma. In the studies reported herein we demonstrate that AXL activation causes p53 functional inactivation in mesothelioma. Specifically, we show that AXL suppresses p53 expression by binding to DNA sequences upstream from the p53 gene, thereby blocking transcription of p53 DNA into RNA. We also show that AXL inhibition by a selective drug inhibits mesothelioma cell viability, migration, and invasion. p53 inactivation attenuates the impact of the AXL inhibitor, providing further support for interplay between AXL and p53 in mesothelioma oncogenesis. These studies demonstrate a novel feedback regulation loop between AXL and p53, and provide a rationale for mesothelioma therapies targeting AXL/p53 signaling.

**Abstract:**

Malignant mesothelioma is a locally aggressive and highly lethal neoplasm. Dysregulation and activation of Gas6/AXL tyrosine kinase signaling are associated with mesothelioma progression, but the mechanisms of these AXL tumorigenic roles are poorly understood. p53 mutants in lung carcinoma upregulate AXL expression by binding and acetylating the *AXL* promoter. Although *TP53* mutations are uncommon in mesothelioma, we hypothesized that these tumors might have alternative feedback mechanisms between AXL and p53. In the current report, we investigated AXL regulation of *TP53* transcription, expression, and biological function in mesothelioma. AXL expression was stronger in mesothelioma than most of the other tumor types from the TCGA gene expression profile dataset. *AXL* knockdown by shRNA induced wild-type and mutant p53 expression in mesothelioma cell lines, suggesting that AXL pro-tumorigenic roles result in part from the suppression of p53 function. Likewise, induced AXL inhibited expression of wild type p53 in COS-7 cells and 293T cells. Immunofluorescence staining showed nuclear colocalization of AXL and p53; however, association of AXL and p53 was not demonstrated in immunoprecipitation complexes. The AXL effects on p53 expression resulted from the inhibition of *TP53* transcription, as demonstrated by qRT-PCR after *AXL* silencing and *TP53* promotor dual luciferase activity assays. Chromatin immunoprecipitation-qPCR and sequencing showed that AXL bound to the initial 600 bp sequence at the 5′ end of the *TP53* promoter. AXL inhibition (shRNA or R428) reduced mesothelioma cell viability, migration, and invasion, whereas *TP53* shRNA knockdown attenuated antiproliferative, migration, and invasive effects of AXL silencing or AXL inactivation in these cells. These studies demonstrate a novel feedback regulation loop between AXL and p53, and provide a rationale for mesothelioma therapies targeting AXL/p53 signaling.

## 1. Introduction

Mesothelioma is an asbestos-associated, locally aggressive, highly lethal, and notoriously chemotherapy-resistant cancer which generally arises from pleural or peritoneal surfaces [1,2]. Conventional chemotherapies and radiation therapy have limited efficacy against mesothelioma. Targeted therapies, including immunotherapies in patients with PD-L1-positive malignant pleural mesothelioma, have shown activity in initial studies [3,4,5,6]. PDL-1 therapy response and efficacy may depend on histologic subtype and grade [7,8,9,10]. To substantially improve survival in mesothelioma patients, novel targets and more effective pharmacological interventions need to be developed. A better understanding of mesothelioma biology—including GAS6/AXL signaling pathways [11]—will likely be crucial in identifying biologically rational targets for novel therapies.

Various studies demonstrate that the AXL receptor tyrosine kinase (RTK) has crucial roles in tumor growth, apoptosis, metastasis, invasion, and drug resistance. Our previous reports implicate AXL overexpression, activation, and regulation of downstream intermediates including PI3K/AKT/mTOR and RAF/MAPK in mesothelioma tumorigenesis [11,12]. AXL also has tumorigenic roles in carcinomas. For example, AXL regulation of miR-374a and miR-548b conferred drug resistance to EGFR inhibitors such as gefitinib in non-small cell lung cancer (NSCLC), and activated AXL is a prognostic marker and potential therapeutic target in NSCLC [13]. AXL activation also impacts immunotherapy and radiotherapy responsiveness in breast cancer [14], and functions as one of the major contributors to lapatinib resistance in HER2-positive breast cancer [15]. AXL inhibition by the small molecular drug R428 prevented metastasis and prolonged survival in breast cancer mouse models [16].

The tumor suppressor p53 regulates cell growth, DNA damage response, and apoptosis. Various studies have shown that more than half of human cancers harbor *TP53* mutations [17], and more than 50% of these *TP53* mutations are missense mutations [18] which have both gain-of-function and loss-of-function properties [19]. MDM2 and p53 form a direct self-feedback regulatory loop at the transcriptional and degradation levels [20].

Recent studies in lung cancer showed that the mutated p53 upregulated AXL at both mRNA and protein levels through histone acetylation on the *AXL* promoter region [21]. In addition, the p53/miR-34a signaling axis has been found to regulate AXL expression in B-cell chronic lymphocytic leukemia [22], and AXL expression regulation by p53 contributes to invasion and drug resistance in colon and breast cancer cells [23]. These observations collectively indicate that p53 negatively regulates AXL transcription. Our previous studies in mesothelioma demonstrated AXL upregulation and activation, and also demonstrated functional inactivation of p53 by the FAK tyrosine kinase [11,24]. Notably, *TP53* genomic mutations are uncommon in mesothelioma [25]. Our recent studies showed that *AXL* knockdown induced p53 expression [26], suggesting that regulatory interactions between p53 and AXL are bidirectional. Thus, we hypothesized that mesotheliomas might similarly depend on feedback loops between AXL and p53, to activate AXL and functionally inactivate p53. In the present report, we show that *AXL* knockdown induces p53 transcription via loss of AXL-mediated repressive effects on the *TP53* promoter. AXL regulated mesothelioma growth, migration, and invasiveness in a p53-dependent manner, suggesting that AXL/p53 signaling warrants clinical evaluation as a therapeutic strategy in mesothelioma.

## 2. Results

### 2.1. AXL is Overexpressed in Mesothelioma, and Correlated with Poor Survival

**Figure 1:**
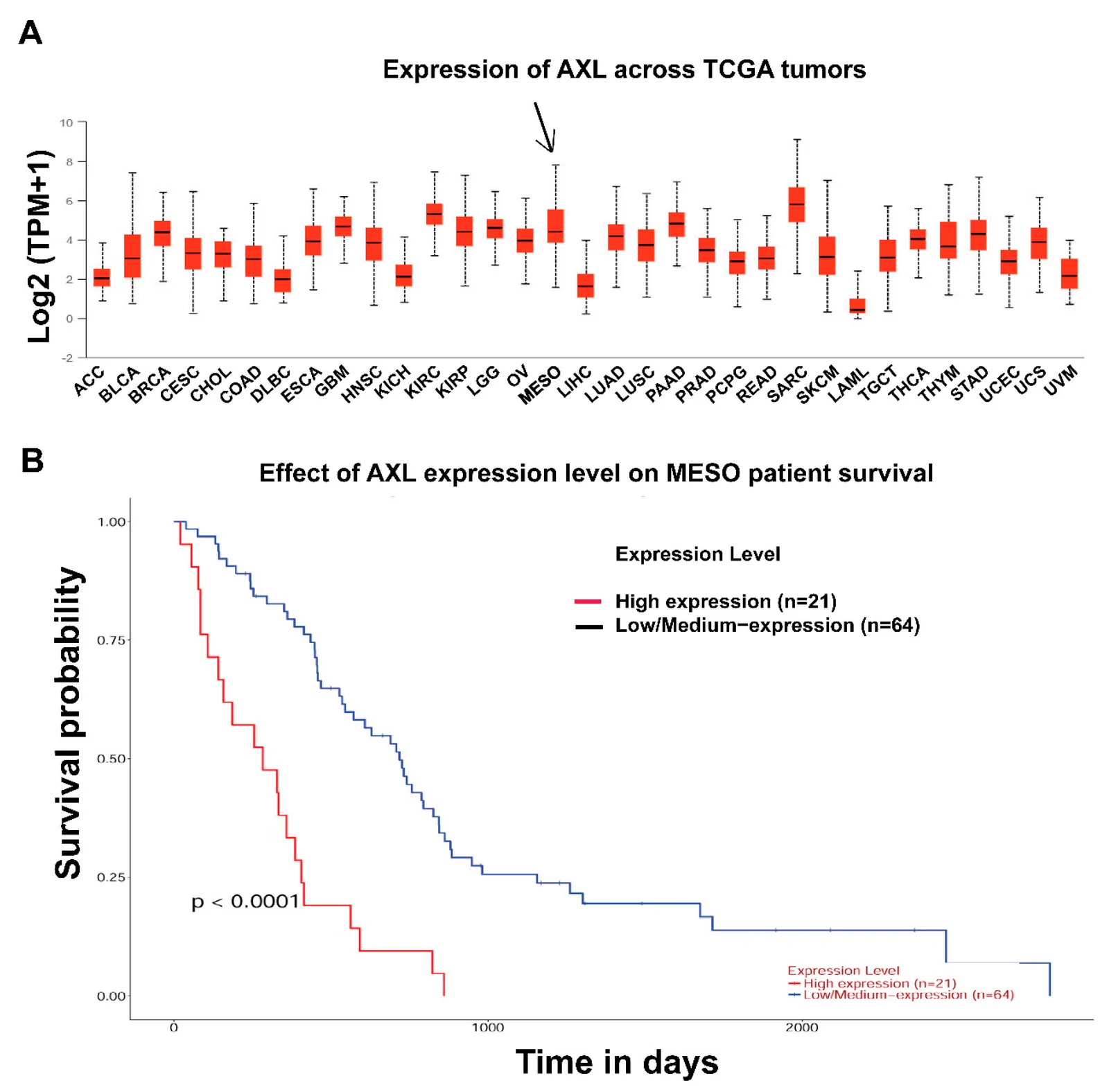
AXL overexpression and survival analysis in mesothelioma. (**A**) TCGA gene expression profiling data analysis on 87 mesotheliomas shows that AXL expression is higher in mesothelioma patient samples compared to other tumor types. (**B**) Survival analysis of TCGA mesothelioma dataset demonstrates that AXL expression level is significantly correlated with overall survival of mesothelioma patients (*p* < 0.0001).

Our previous published data had demonstrated overexpression and activation of AXL in mesothelioma cell lines and biopsy materials, which regulates tumor proliferation via PI3K/AKT/mTOR and RAF/MAPK signaling pathways, and AXL expression was stronger in mesothelioma than in the other tumor types [11]. Herein, we firstly analyzed AXL expression between mesothelioma and other tumor types by using the published TCGA expression profiling data on mesothelioma [27]. AXL expression analysis in 87 mesothelioma patients showed that AXL expression was stronger in mesothelioma (MESO) than in most cancer types, including ovarian cancers, NSCLC, colon adenocarcinoma, and so on (Figure 1A and Figure S1A), confirming our previous immunoblotting observations [11]. TCGA profiles showed that AXL was more highly expressed in biphasic mesothelioma and sarcomatoid mesothelioma than in epithelioid mesothelioma and diffuse malignant mesothelioma (Figure S1B), which is also consistent with our previous immunoblotting findings that AXL expression is strongest in mesothelioma containing spindle-cell components [11]. About 24.7% of mesothelioma patients in the TCGA dataset were in the AXL high expression group (Figure S1B). Furthermore, using the TCGA mesothelioma dataset [27], we analyzed the prognostic value of AXL expression in mesothelioma patients. High AXL expression in mesothelioma was correlated with reduced overall survival (*p* < 0.0001) (Figure 1B). Histologic subtype analyses further showed that higher AXL expression correlated with poorer overall survival in biphasic (*p* = 0.0023) and epithelioid (*p* = 0.0032) mesotheliomas (Figure S1C). There were too few TCGA cases of sarcomatoid or diffuse malignant mesothelioma to permit informative analyses of prognostic relevance within these histologic subgroups.

### 2.2. AXL Regulates p53 Protein Expression in Mesothelioma

**Figure 2:**
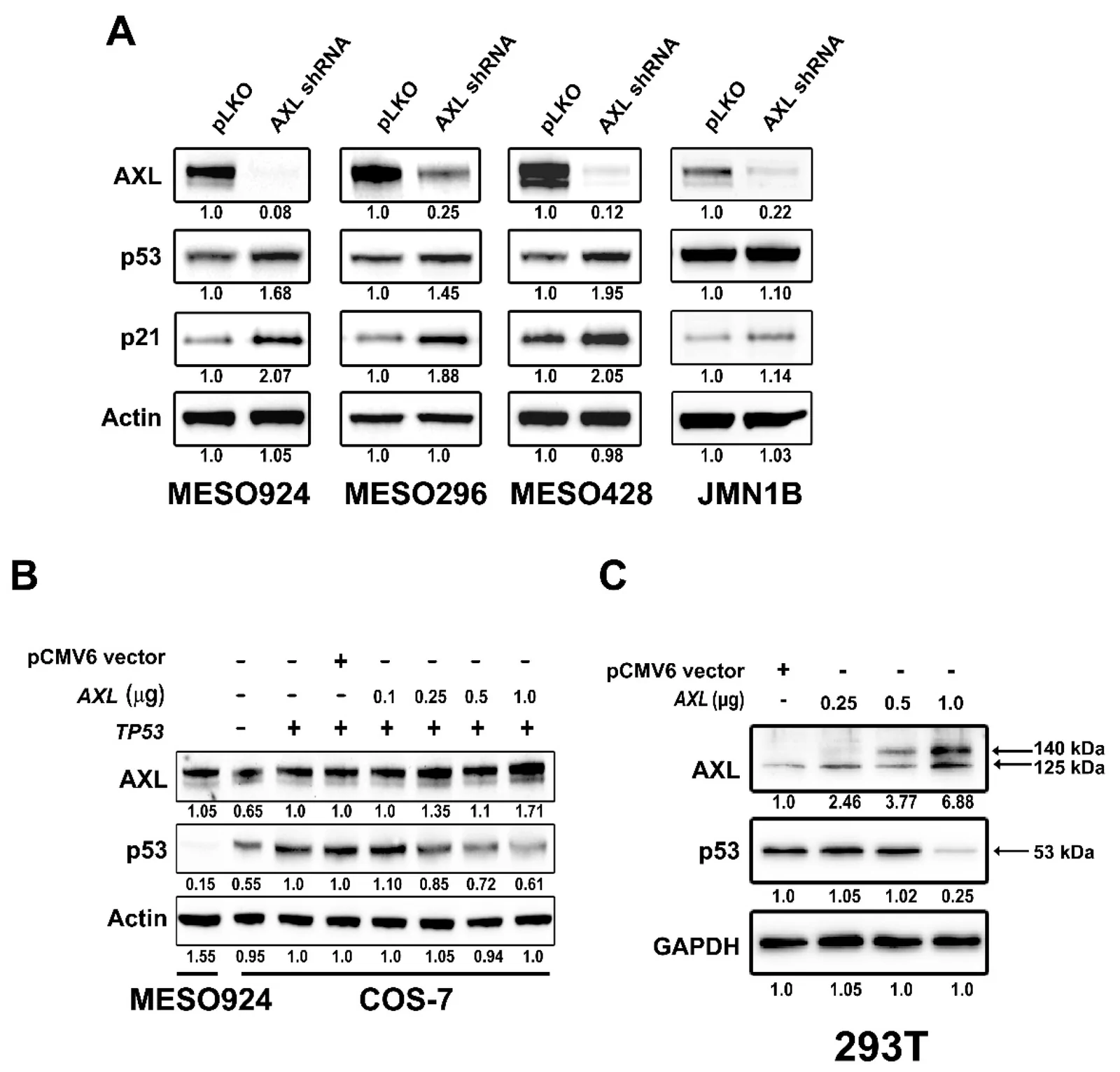
AXL regulates p53 protein expression. (**A**) Immunoblotting evaluation of the expression of AXL, p53, and p21 in MESO924, MESO296, MESO428, and JMN1B cells at 96 h post-infection with lentiviral *AXL* shRNA. Actin stains show equivalence of lane loading. Expression quantitations of AXL, p53, p21, and actin are normalized to control infections using empty vector pLKO. (**B**) Immunoblotting shows dose-dependent AXL inhibition of p53 expression in COS-7 cells 48 h after cotransfection of *TP53* (1 µg) with varying amounts of *AXL*. pCMV6 (1 µg) vector lane is a control, and MESO924 is a positive control for AXL expression. Protein expression quantitations are normalized to control transfections using empty vector pCMV6. (**C**) Immunoblotting shows dose-dependent AXL inhibition of p53 expression in 293T cells 48 h after transfection with varying amounts of *AXL*. pCMV6 (1 µg) vector lane is a control. GAPDH stain is a loading control. Expression quantitations of AXL and p53 are normalized to control transfections using empty vector pCMV6. The whole blot (uncropped blots) show in the Figure S3.

*AXL* gene expression was stably silenced by lentivirus-mediated shRNA in three wild type p53 mesothelioma cell lines (MESO924, MESO296, and MESO428) and one mutated p53 mesothelioma cell line JMN1B, resulting in more than 90% inhibition of AXL when assessed 96 h after transduction (Figure 2A). The *AXL* shRNA-mediated knockdown resulted in upregulation of p53 and p21 in p53 wild type cell lines, and a mild increase in the expression of p53 and p21 in p53 mutant JMN1B (Figure 2A). Using MESO924 as a positive control, AXL inhibited expression of p53 after *AXL* and *TP53* expression constructs were cotransfected into COS-7 cells (Figure 2B). Similarly, p53 protein expression was inhibited in 293T cells by overexpression of *AXL* in a DNA dose-dependent manner (Figure 2C). The expression quantifications of AXL, p53, or p21 were normalized to control empty vector pLKO or pCMV6.

The above-mentioned findings indicate that AXL regulates mesothelioma proliferation, in part, by p53 dysregulation. Thus, we used the TCGA mesothelioma dataset to check whether AXL expression correlated with *TP53* expression [27]. However, *AXL* vs. *TP53* transcript expression from the TCGA mesothelioma series did not show a clear correlation. Thus, instead of comparing transcript expression, we used immunoblotting to evaluate the protein expression of AXL and p53 in 10 mesothelioma cell lines and in eight clinical mesotheliomas tissue samples (Figure S2). There was an inversely proportional relationship between AXL and p53 in mesotheliomas with nonmutant *TP53*, i.e., cases with higher AXL expression had lower p53 expression. MESO507 and sample 7 from mesothelioma cell lines and tumor tissues, respectively, which harbored *TP53* point mutation did not show this relationship (Figure S2).

### 2.3. No Protein Interaction is Detected between AXL and p53

**Figure 3:**
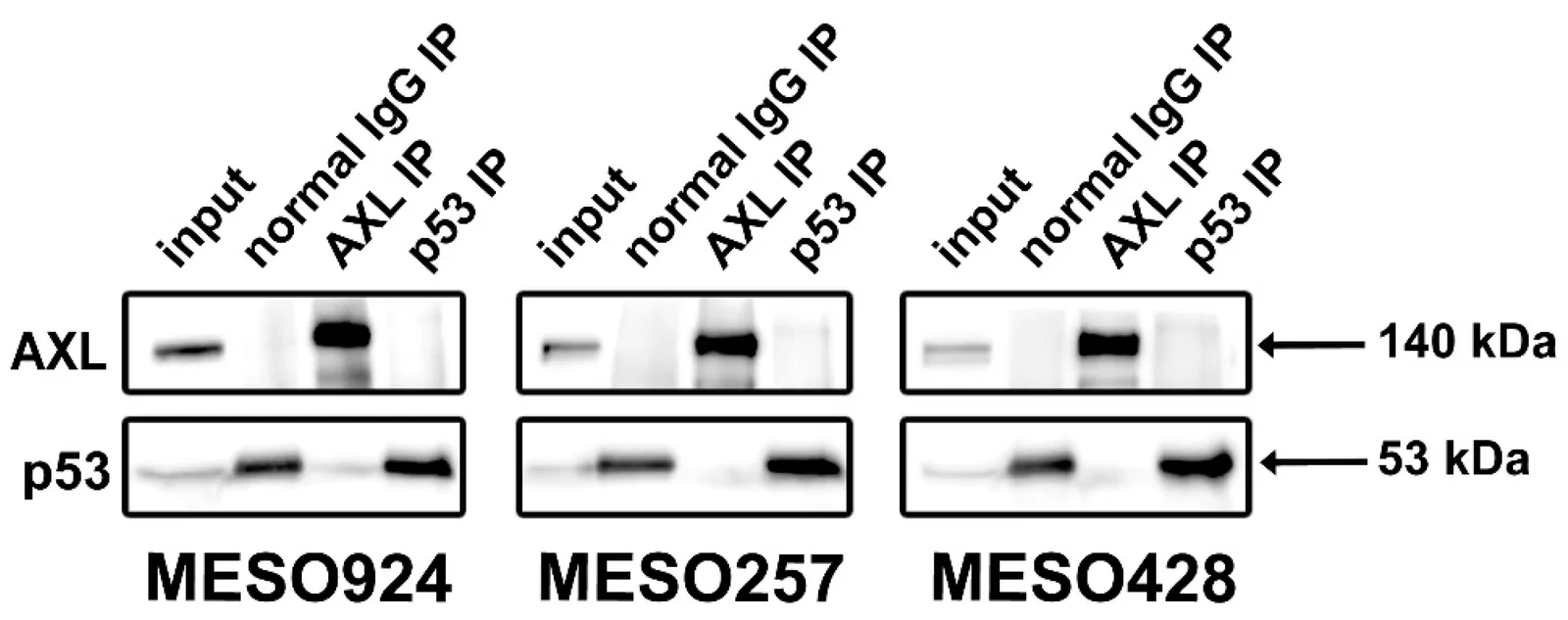
AXL/p53 Immunoprecipitations followed by AXL and p53 immunoblotting in mesothelioma cell lines (MESO924, MESO257, and MESO428) do not show interaction between AXL and p53. The whole blot (uncropped blots) show in the Figure S3.

We next investigated whether there was an interaction between AXL and p53 in mesothelioma cell lines (MESO924, MESO257, and MESO428) using AXL and p53 immunoprecipitations, followed by AXL and p53 immunostaining (Figure 3). The p53 immunoprecipitations did not pull down an AXL 140 kDa band in three mesothelioma cell lines, and the AXL immunoprecipitations did not pull down a p53 53 kDa band in these cells, as compared to the control IP. Normal mouse IgG immunoprecipitations were used as controls, and nonspecifically bound some p53, even though protein lysates were preabsorbed for 30 min using 20 μL of protein G beads (Figure 3).

### 2.4. AXL Colocalization with p53 in the Nucleus

**Figure 4:**
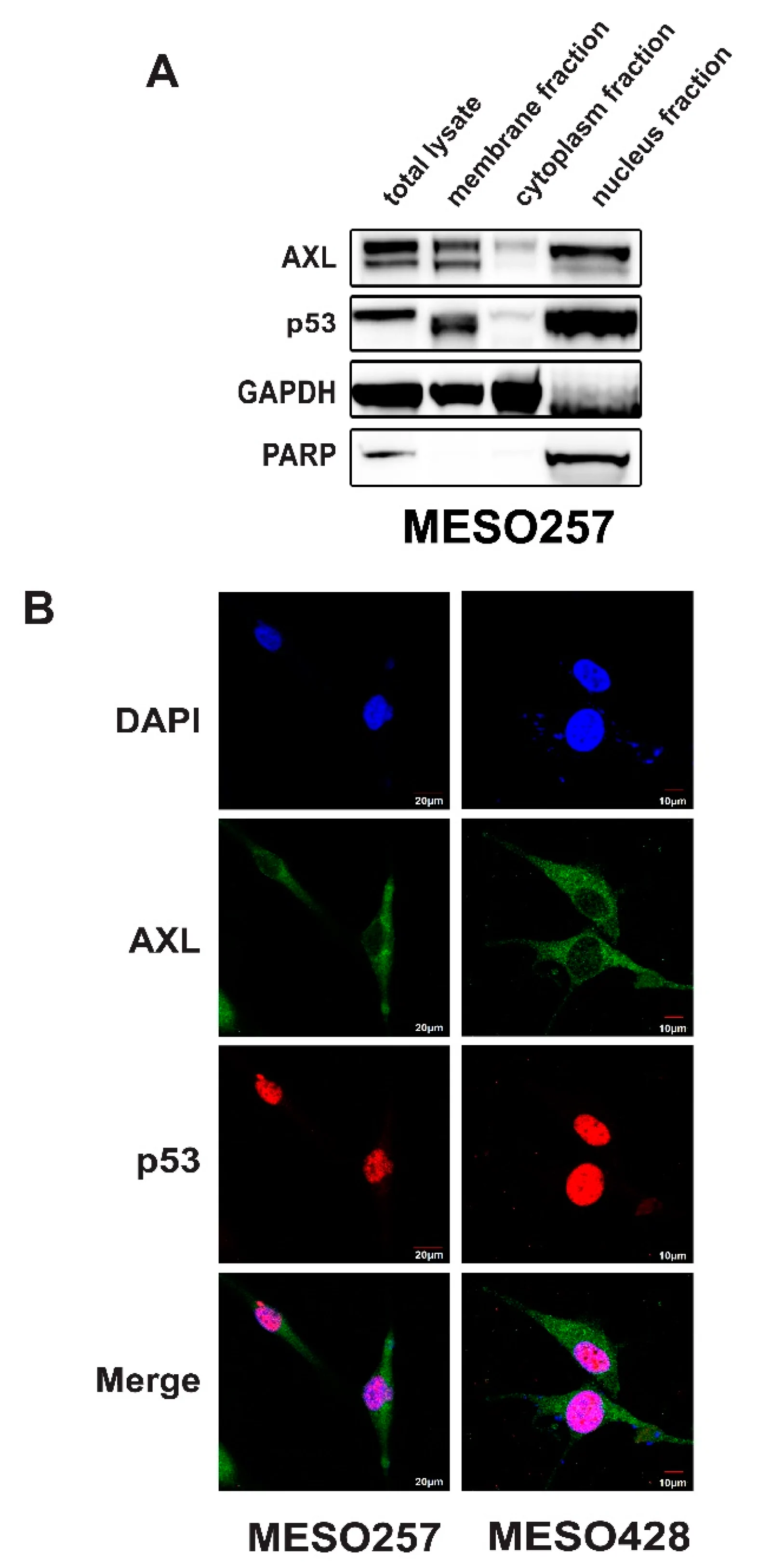
AXL colocalization with p53 in the nucleus. (**A**) Reproduced from Fletcher et al. [24], licensed under CC BY-NC 4.0. Nuclear localization of AXL and p53 was evaluated in MESO257 by immunoblotting. Poly (ADP-ribose) polymerase (PARP) is a nuclear localization control, and GAPDH is a cytoplasmic control. The whole blot (uncropped blots) show in the Figure S3. (**B**) Cell distribution of AXL and p53 in MESO257 and MESO428 by immunofluorescence staining. The green stain represents AXL expression and red stain represents p53 expression. The color of image mergence of AXL (green) and p53 (red) stains showed orange. All the assays were performed from triplicate experiments.

Nuclear colocalization of AXL and p53 was evaluated using cell component isolation and immunofluorescence staining (Figure 4). Immunoblotting of cell component fraction separation in MESO257 showed that AXL and p53 colocalized in the nucleus. Compared to PARP immunostains between total protein lysates and nucleus fraction, nucleus fraction samples were overloaded, and showed strong AXL and p53 bands (Figure 4A). Immunofluorescence in MESO257 and MESO428 showed that AXL (green color) expression was predominantly cell membrane, cytoplasmic, and nuclear, whereas p53 (pink color) expression was nuclear and cytoplasmic, with colocalization of AXL and p53 (Figure 4B).

### 2.5. AXL Negatively Regulates TP53 Transcription in Mesothelioma

**Figure 5:**
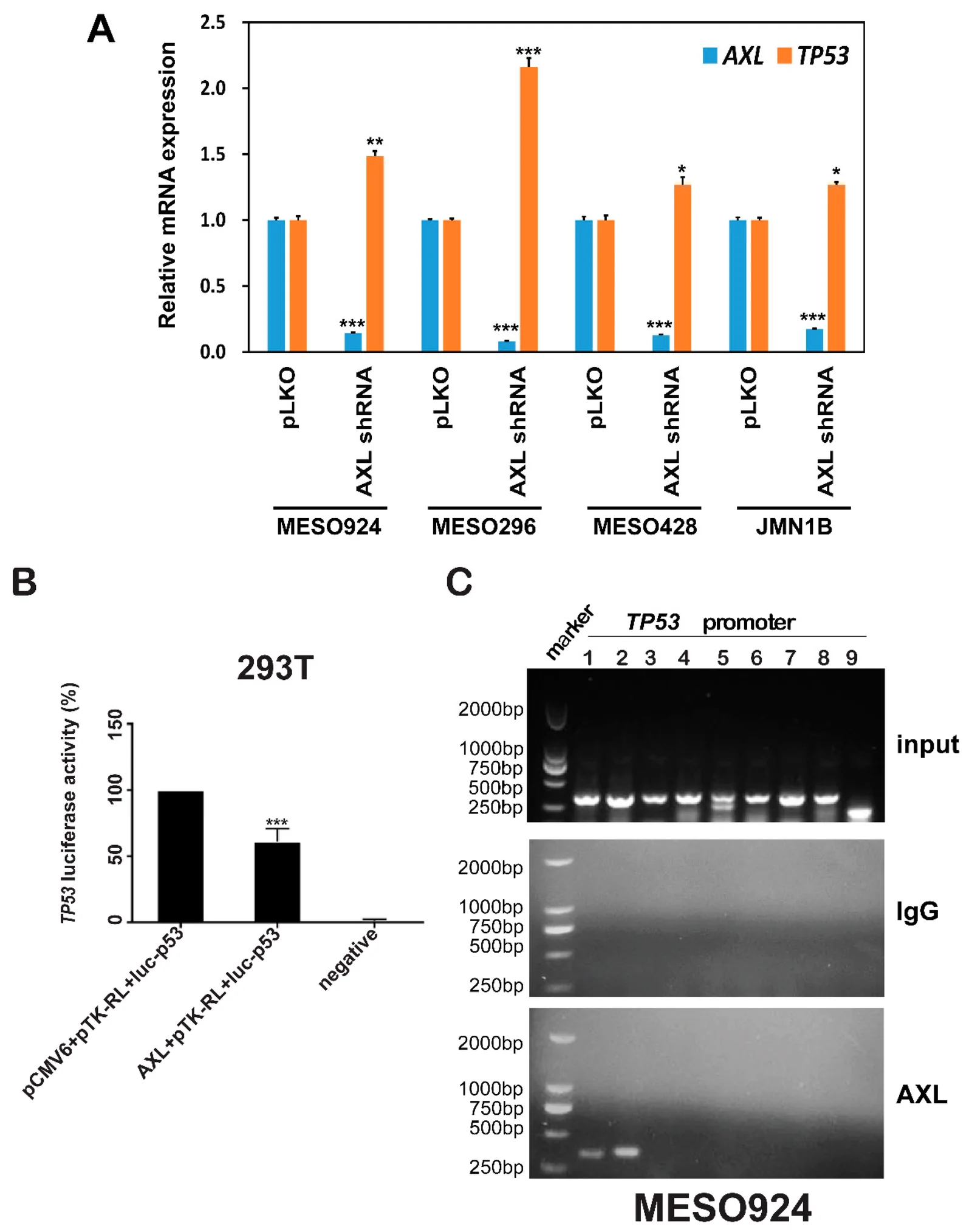
AXL negatively regulates *TP53* transcription. (**A**) Quantitative RT-PCR evaluations of *AXL* (blue bars) and *TP53* (green bars) transcripts at 72 h after infection of MESO924, MESO296, MESO428, and JMN1B cells with lentiviral *AXL* shRNA constructs. The comparative Ct (cycle threshold) method was used to determine RNA expression, which was normalized to pLKO of each cell lines in triplicate assays. Statistically significant differences are presented as * *p* < 0.05, ** *p* < 0.01, *** *p* < 0.001. (**B**) AXL expression inhibits *TP53* promoter activity: *TP53* luciferase reporter plasmid *luc-p53* (0.25 µg) and *Renilla* luciferase reporter plasmid *pTK-RL* (0.005 µg) were cotransfected with pcDNA3 empty vector or *AXL* (0.25 µg) into 293T cells. Transfected cells were harvested at 48 h, and assessed using a Dual-Luciferase Reporter Assay System. Transfection efficiencies were normalized to the *pTK-RL* luciferase plasmid, and *TP53* luciferase activities were normalized to the pCMV6 vector (100%). The data represent the mean values (± s.d.) from quadruplicate cultures. All the assays were performed from triplicate experiments. Statistically significant differences are presented as *** *p* < 0.001. (**C**) Chromatin immunoprecipitation-qPCR. *TP53* promoter was amplified by nine pairs of primers and evaluated by 1% agarose gel. Chromatin immunoprecipitation showed that AXL directly binds to the first 600 bp at the 5′ end promoter region of *TP53*. Mean ± S.D.; *n* = 3; IgG is a negative control.

As AXL knockdown induced p53 protein expression (Figure 2A), we further analyzed *TP53* transcripts after AXL silencing (Figure 5A). mRNA levels of *TP53* and *AXL* were evaluated by qRT-PCR in three wild type p53 mesothelioma cell lines (MESO296, MESO428, and MESO924) and one mutated p53 mesothelioma cell line JMN1B, at 96 h post-transduction by lentiviral *AXL* shRNA (Figure 5A). qRT-PCR demonstrated that the *TP53* transcript was increased after *AXL* knockdown (Figure 5A). In dual-luciferase reporter assays, AXL inhibited *TP53* promoter activity by 40% (Figure 5B).

To demonstrate and localize AXL binding sequences in the *TP53* promoter, chromatin immunoprecipitation (ChiP) assay was performed in MESO924 (Figure 5C). Nine pairs of primers of the *TP53* promoter were designed to amplify the DNA isolated from AXL immunoprecipitation (Table S1). *TP53* PCR products were obtained using two pairs of primers that generate amplicons for the first ≈ 600 bp sequence at the end of 5′ in the *TP53* promoter: TCGCCCTGCCCCGTACAGTCCCGTTTGTGACAGGAACACTTGGACTACACTTTTTCCATCTCCACGAAGGACTTAAATGTGCAGCATTGATGAAGAGGCAGTTAGCCCCAGTCCTTACCATTTCTGCATATTCCACCTGCTCCCCCTCATGGTACAGCTCTGGGGGCAGGTTATAAATTCGCAAGATGTTATCAGCACTATTGGTCAAGATGCAGGAACCGTCAGGAGCCCTAGAAACAGGGGAGAGTTAGAAAGCTGGCCAGACCTATGCTTTTCAAGTGTAGGGCTAGGGCTGAGCCTGCCTCTGGGGTAGGTAAGCACCCCTG.ATCCTTGAGGGAAGTAGACGACACAAACTGCTAGATAAAATGTAAGCTCATTCTAAAAGGGCTACGTGCCGCTTCTCCCAGCTCTGGGGCATCCCTCTCCTAGAAAACTGGACTGTTTTACAGTGAAAATCTCGGGGGTGGTCAGCTCCCTGCCCCGTTGTTATCCTTACCACTTACAGCCTTTCAAGAAGTTCTCAGGTTGGGTGCTGAACTCTGACCAGGAACCACTGAGAAATCGAGGCAGCTGGGAGAAGCTGTAGTTCCAAGCGCTGAA.

### 2.6. AXL Regulates Mesothelioma Migration, Invasiveness, and Proliferation through p53

**Figure 6:**
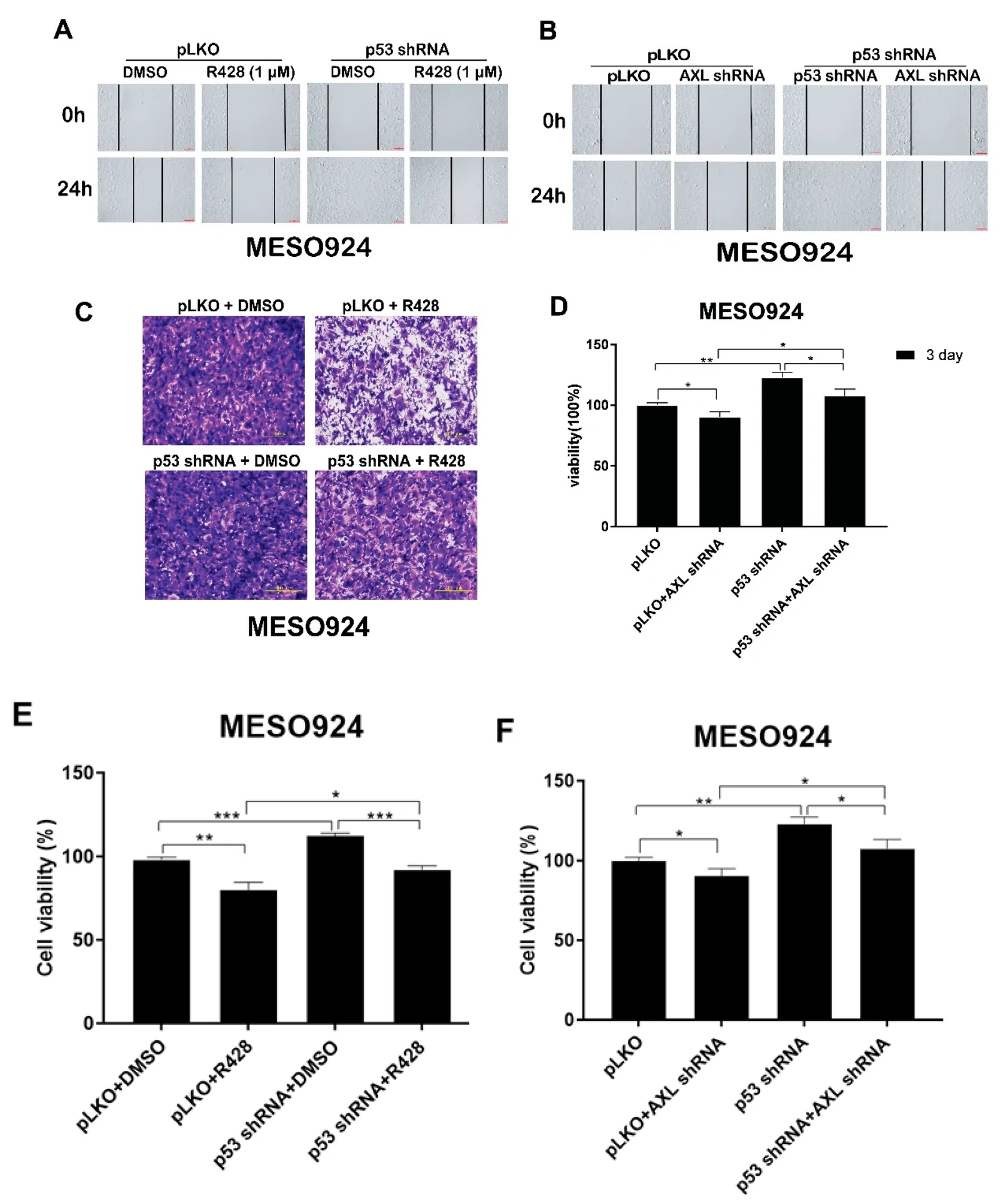
AXL regulates mesothelioma migration, invasiveness, and proliferation through p53. (**A**,**B**) R428 (1 µM) treatment or lentiviral shRNA-mediated AXL knockdown inhibited migration of MESO924, but *p53* shRNA knockdown attenuated antimigration effects of R428 treatment in MESO924, as assessed by in vitro wounding assays. (**C**,**D**) R428 (1 µM) treatment inhibited invasive effects of MESO924 cells, but *p53* shRNA knockdown attenuated anti-invasive effects of R428 treatment in MESO924, as assessed by transwell matrigel assays. Data were normalized to the empty lentivirus infections or DMSO and represent the mean values (±s.d.) of triplicate cultures. All the assays were performed from triplicate experiments. Statistically significant differences are presented as * *p* < 0.05, ** *p* < 0.01. (**E**,**F**) Cell viability was evaluated by MTT assay in MESO924 at day 3 after infection with lentiviral *AXL* shRNA, but *p53* shRNA knockdown attenuated antiproliferative effects of AXL silencing or AXL kinase inhibition (R428) in MESO924. Data were normalized to the empty lentivirus infections or DMSO and represent the mean values (±s.d.) of triplicate cultures. All the assays were performed from triplicate experiments. Statistically significant differences are presented as * *p* < 0.05, ** *p* < 0.01, *** *p* < 0.001.

Our published data have demonstrated that AXL is activated and overexpressed in mesothelioma cell lines (MESO924, MESO296, MESO257, and MESO428) and mesothelioma tumor tissues [11,12]. Wound-healing assays in AXL-phosphorylated MESO924 cells demonstrated that AXL inhibition (R428) or *AXL* shRNA knockdown resulted in greater inhibition of wound closure at 24 h than in control cells treated with DMSO or infected with empty lentiviral vector (Figure 6A,B). However, *TP53* shRNA-mediated knockdown resulted in complete wound closure as compared to the control cells infected with empty lentiviral vector (Figure 6A,B). Cell migration was less inhibited by R428 or *AXL* shRNA after p53 knockdown than after AXL silencing or inactivation. MESO924 cells with p53 knockdown showed similar results to the control cells treated with DMSO or infected with empty lentiviral vector (Figure 6A,B).

Assays were performed in MESO924 to evaluate the effects of AXL inhibition and p53 knockdown on mesothelioma cell invasion. Transwell matrigel assays demonstrated that AXL inhibition (R428) resulted in greater inhibition of invasiveness at 24 h than those of control cells treated with DMSO and infected with empty lentiviral vector (Figure 6C,D), whereas *p53* shRNA knockdown increased invasiveness as compared to the control cells infected with empty lentiviral vector (Figure 6C,D). Cell invasion was less inhibited by R428 after p53 knockdown. MESO924 growth after p53 knockdown was equivalent to that of control cells treated with DMSO and infected with empty lentiviral vector (Figure 6C,D).

AXL knockdown or kinase inhibition in MESO924 resulted in ≈30% inhibition of cell viability (Promega CellTiter-Glo assay; Madison, WI, USA) at 3 days after AXL silencing or treatment with R428, compared with the empty vector or DMSO control (Figure 6E,F). *p53* shRNA-mediated knockdown had little effect on cell viability as compared to the control cells infected with empty lentiviral vector (Figure 6E,F). However, *p53* shRNA knockdown decreased the antiproliferative effects of AXL kinase inhibition or AXL knockdown in MESO924, and showed similar results to the control cells treated with DMSO or infected with empty lentiviral vector (Figure 6E,F).

## 3. Discussion

Currently available therapies, including surgery, radiation, chemotherapy, target therapies, and immunotherapies, have done little to improve the outcome for mesothelioma patients. Advances in understanding mesothelioma biology and tumorigenesis will likely enable development of more effective therapeutics. Our previous studies indicate that activation of Gas6/AXL signaling plays essential functional roles in mesothelioma tumorigenesis [11,12]. Furthermore, inhibition of PI3K/AKT signaling is associated with aberrant MDM2-p53 interaction in mesothelioma [28]. Cotargeting of FAK-p53 and MDM2-p53 interactions exhibited antiproliferative effects in mesothelioma [24], suggesting that p53 functional inactivation is a key event in mesothelioma development, as genomic *TP53* mutations are not frequent in mesothelioma [29]. These findings suggest that p53 has a tumor suppressor function and is inactivated by multiple signaling pathways including RTK in mesothelioma. The studies reported herein provide insights into mesothelioma biology and identify novel interactions between AXL and p53 that might provide opportunities for novel therapeutic strategies.

The current results and our previous studies show that AXL expression is stronger in mesothelioma, particularly those with spindle-cell components [11], than in most other cancer types. Further, high AXL expression in mesothelioma is significantly associated with poorer survival (Figure 1 and Figure S1). These findings indicate that high AXL expression identifies a subset of mesotheliomas for which novel therapies are a particularly urgent need.

Previous evidence demonstrated that p53 mutants induce upregulation of *AXL* transcript via direct binding to *AXL* promoter, and p53 mutant knockdown reduced acetylation of *AXL* promoter histones in lung cancer [21], indicating that p53 regulates *AXL* transcription. Although no direct evidence shows this correlation between AXL and p53 in the published mesothelioma TCGA dataset, there seems to be a p53 negative regulation by AXL in tested mesothelioma cell lines and tumor tissues (Figure S2). p53 expression is regulated in mesothelioma at both transcriptional and protein levels by various mediators such as MDM2 [30], FAK [24], HDAC [31], PI3K/AKT intermediates [28], and so on. Therefore, it can be explained that there is no robust correlation between AXL and *TP53* in mesotheliomas (Figure S2). However, in our current studies, AXL knockdown resulted in p53 upregulation in three wild-type p53 mesothelioma cell lines (MESO924, MESO296, and MESO428) and one p53 mutant mesothelioma cell line (JMN1B), and overexpression of AXL inhibited p53 expression after cotransfection of *AXL* and *TP53* (wild type) expression constructs in COS-7 cells or transfection with *AXL* construct alone in the 293T cell line. These studies suggest that AXL mediates p53 expression irrespective of p53 mutational status (Figure 2 and Figure 5A). Thus, we hypothesized that there may be a self-feedback regulatory loop between AXL and p53, namely, p53 negatively regulates transcription and expression of *AXL*, and AXL negatively regulates transcription and expression of *TP53.*

To address this hypothesis, we first investigated whether AXL complexed with p53 protein in three mesothelioma cell lines (MESO924, MESO257, and MESO428). However, AXL and p53 co-immunoprecipitations showed no interaction between the AXL and p53 proteins (Figure 3). We next analyzed whether AXL colocalized with p53. Cell component separation and immunofluorescence stains demonstrated that AXL and p53 have nuclear colocalization (Figure 4A,B). Various studies have shown that nuclear translocation of RTKs (full-length or truncated) by c-secretase cleavage of the full-length receptor, shedding, alternative splicing, or alternative translation initiation, including EGFR, ERBB2, FGFR1, KIT, and AXL, regulates transcription [32,33,34,35,36,37]. A recent report found that AXL, but not the other two members of the same family MERTK or TYRO3, is cleaved by α- and γ-secretases in cancer cell lines to generate an AXL intracellular isoform. These AXL isoforms contain a nuclear localization sequence harboring a basic HRRKK motif, which is released from the plasma membrane and translocated into the nucleus [37]. The current cell component separation showed that the full-length AXL can also translocate into the nucleus in mesothelioma (Figure 4A). These findings suggest that regulatory relationships between AXL and p53 result from their colocalization in the nucleus.

**Figure 7:**
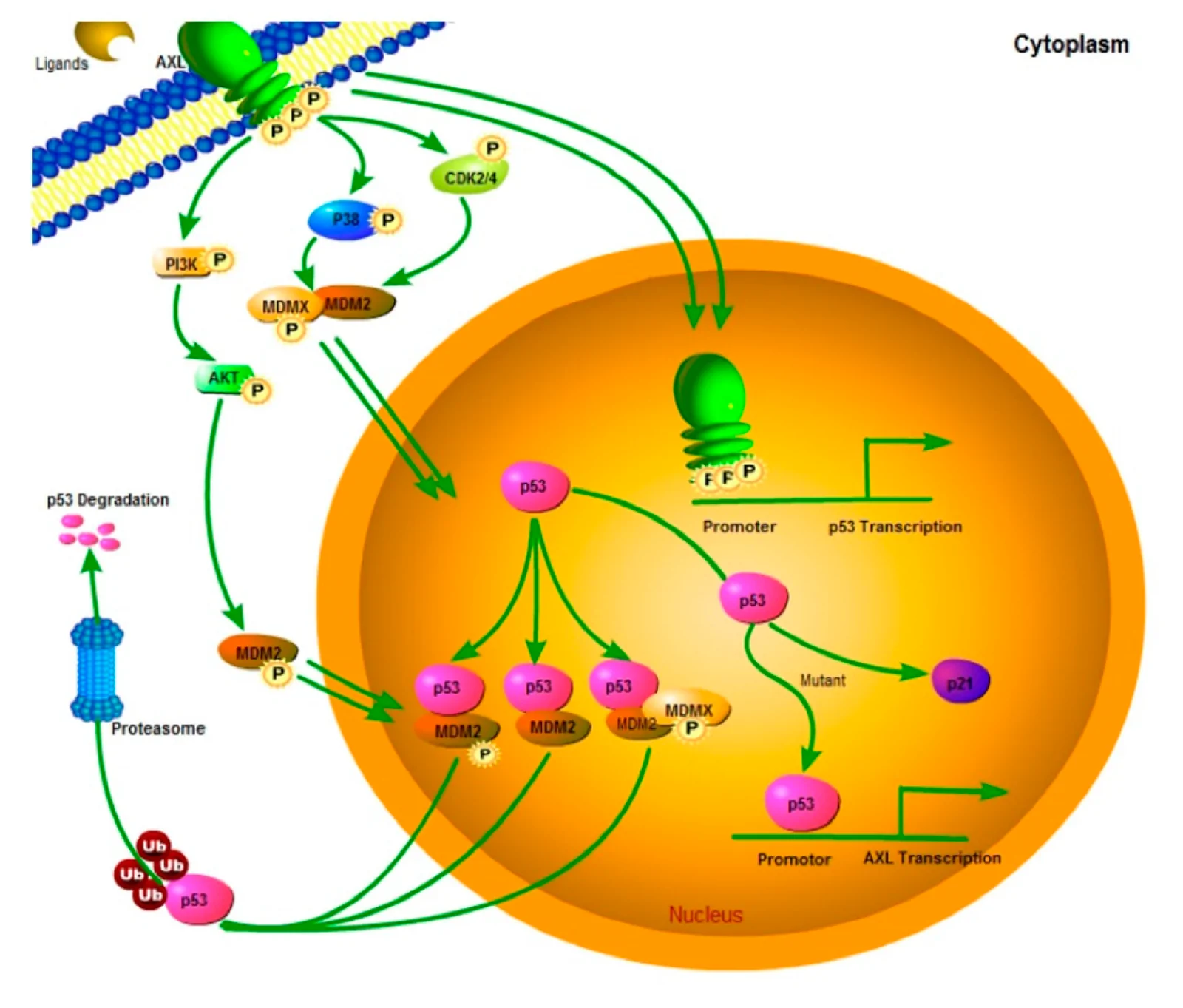
Model of AXL-mediated p53 turnover and cell growth. AXL can promote cell growth and migration through signaling cascades such as AKT that can activate ubiquitin E3-ligases such as MDM2 to maintain low p53 levels. Nuclear AXL stabilizes a MDM2-MDMX-p53 complex, leading to p53 polyubiquitination and subsequent p53 degradation by nuclear or cytoplasmic proteasomes. At the same time, nuclear AXL binds to the *TP53* promoter to regulate the p53 transcription and expression, and p53 binds to the *AXL* promoter to regulate the AXL transcription and expression. This regulatory connection between AXL and p53 is a direct self-feedback regulatory loop.

Although AXL and p53 both localize to the nucleus, we did find demonstrable complexing between these proteins. Therefore, we evaluated whether AXL regulates *TP53* transcription. qRT-PCR demonstrated that *AXL* shRNA knockdown upregulated *TP53* transcripts in each of three wild-type *TP53* mesothelioma cell lines (MESO924, MESO296, and MESO428), and one *TP53*-mutant mesothelioma cell line (JMN1B) (Figure 5A). Furthermore, dual luciferase reporter assays showed that AXL inhibited *TP53* promoter transcriptional activity (Figure 5B). ChiP-PCR, agarose gel electrophoresis, and sequencing in MESO924 illustrate that AXL binds to a 600 bp sequence at the 5′ end of the *TP53* promoter (Figure 5C). These data demonstrated that AXL negatively regulated *TP53* transcription and p53 protein expression through direct binding to the *TP53* promoter. Further evaluations of the nuclear localization of AXL and its binding to *TP53* promoter region will be performed in detail in the near future. These studies will include identifying the AXL binding domain as well as the exact binding site of AXL to the *TP53* promoter. This will be followed by introducing artificial mutations to the sites to inhibit this binding. Interestingly, a recent finding showed that AXL suppressed p53 in melanoma through stabilization of the MDMX-MDM2 complex. AXL activation stabilized MDMX via a post-translational modification that involved phosphorylation of MDMX [38]. Collectively, these data demonstrate that AXL negatively regulates p53 expression by at least two mechanisms: (1) *TP53* transcription inhibition, irrespective of p53 mutation status; (2) stabilization of the MDMX-MDM2 complex. Additionally, previous studies demonstrated that p53 mutants induced upregulation of AXL by direct binding of p53 mutants to *AXL* promoter [21]. Those data further support the presence of a feedback regulation loop between AXL and p53. PI3K/AKT regulates MDM2-p53 cell-cycle check points in mesothelioma [28], and MDM2 and p53 form a direct self-feedback regulatory loop at the transcriptional and degradation levels [20]. Our current results and previous findings [21,28,38] were used to suggest a feedback regulation model between AXL, p53, and MDM2, as shown in Figure 7.

Finally, to investigate AXL/p53 signaling roles in tumor migration, invasion, and cell viability, we assessed mesothelioma cell line MESO924 (AXL-activated with wild-type p53) after AXL inhibition by R428 or shRNA in the absence or presence of *p53* shRNA (Figure 6). *AXL* shRNA knockdown or kinase inhibition with R428 inhibited cell viability, migration, and invasion in MESO924 (Figure 6), which is consistent with our previous findings [11]. *p53* shRNA knockdown attenuated these antiproliferative, antimigration, and anti-invasive effects of AXL silencing or inhibition (Figure 6). The results indicate that AXL plays a crucial role in tumor growth and migration in mesothelioma by regulating p53 expression. These novel findings suggest that therapeutic strategies targeting AXL/p53 signaling might be effective in mesothelioma.

## 4. Materials and Methods

A number of the methods described below and mesothelioma cell lines have been described previously [24,28,30,39].

### 4.1. Antibodies and Reagents

Polyclonal goat antibody to AXL (#sc-1096), and monoclonal mouse antibodies to AXL (#sc-166269), p53 (#sc-126), and p21 (#sc-6246) were purchased from Santa Cruz Biotechnology (Santa Cruz, CA, USA). Polyclonal antibody to GAPDH (#10494-1-AP) was from Proteintech (Wuhan, Hubei, China). Anti-rabbit HRP-IgG (#7074) and anti-mouse HRP-IgG ((#7076) were from Cell Signaling Technology (Pudong, Shanghai, China). Donkey anti-goat HRP-IgG (#sc-2020), Protein A (#sc-2001)/Protein G (#sc-2002) beads were obtained from Santa Cruz Biotechnology. Lipofectamine, Plus reagent, and Trizol were obtained from Invitrogen Life Technologies (Carlsbad, CA, USA). Monoclonal mouse antibody to actin (#A4700), Crystal violet, and polybrene were from Sigma-Aldrich (St Louis, MO, USA). The Bio-Rad iScriptTM cDNA synthesis Kit and iQ SYBR green supermix were obtained from Bio-Rad Laboratories (Hercules, CA, USA). Lentiviral shRNA constructs were from The RNAi Consortium (TRC, Cambridge, MA, USA), and included *AXL*: GCTGTGAAGACGATGAAGATT; and *p53*: CTTCGACTATCTCAAACTCCT. *AXL* and *p53* expression plasmid was purchased from Origene Technologies, Inc. (Rockville, MD, USA). The luciferase reporters of *luc-p53* (−2.4 kb) promoter has been described previously [40,41]. R428 (also named Bemcentinib, which has been used in clinical trials) was obtained from Selleck (Houston, TX, USA). The inhibitor was reconstituted in DMSO.

### 4.2. Mesothelioma Cell Lines and Frozen Tumor Specimens

Mesothelioma cell lines were established from surgical materials from previously untreated patients, as reported previously [11,24]. MESO924, MESO257, MESO1401, MESO542, MESO507, and MESO346 cell lines were established from epithelial-type mesotheliomas, MESO296 and MESO647 from mixed-histology mesotheliomas, and MESO188 and MESO428 from a spindle-cell mesothelioma. MESO924, MESO296, MESO428, MESO188, MESO1401, MESO542, MESO346, MESO647, and MESO257 harbor wild-type p53. MESO507 harbored *TP53* S241F missense mutation. An additional mesothelioma cell line, JMN1B, was established from an epithelial-type mesothelioma [42], had been found to contain *TP53* G245S missense mutation. Derivation of each cell line from the corresponding surgical specimen was corroborated by STR profiling. All mesothelioma frozen tumor specimens were discarded tissues, obtained from Brigham and Women’s Hospital. The studies were conducted in accordance with recognized ethical guidelines (U.S. Common Rule), and approved by Brigham and Women’s Hospital and Zhejiang Sci-Tech University Institutional Review Boards.

### 4.3. Immunoblotting

Cell lysates were prepared using lysis buffer (1% NP-40, 50 mM Tris-HCl (pH 8.0), 100 mM sodium fluoride, 30 mM sodium pyrophosphate, 2 mM sodium molybdate, 5 mM EDTA, 2 mM sodium orthovanadate, 10 mg/mL aprotinin, 10 mg/mL leupeptin, and 1 mM phenylmethylsulfonyl fluoride). Lysate protein concentrations were determined using a Bio-Rad protein assay (Bio-Rad Laboratories, Hercules, CA, USA) after Lysates were cleared by centrifugation at 14,000 rpm for 30 min at 4 °C. According to the manufacturer’s protocol, nuclear, membrane, and cytoplasmic fraction lysates were prepared using a Qproteome Cell Compartment Kit (Qiagen Inc., Valencia, CA, USA). Electrophoresis and immunoblotting were performed as described previously [43]. The hybridization signals were detected by chemiluminescence (ECL, Amersham Pharmacia Biotechnology, Marlborough, MA, USA) and captured using an ImageQuant LAS4000. Linear capture quantitation of immunoblotting chemiluminescence signals, using an ImageQuant LAS4000. Intensity values are standardized to the pLKO/pCMV6 control.

### 4.4. Immunoprecipitation

A total of 1 mg of protein lysates was preabsorbed for 30 min using 20 μL of protein G beads at 4 °C. Two micrograms of p53 or AXL primary antibodies were added and incubated with the lysates for 2 h at 4 °C. Then, 20 µL of sepharose-protein G was added and rocked overnight at 4 °C. The sepharose beads were washed three times with 750 µL of IP buffer (10 min/each time) and once with 750 µL 10 mM Tris-Cl buffer (pH 7.6). Loading buffer (20 μL) was added to the beads and boiled for 5 min at 95 °C. Three independent experiments were performed.

### 4.5. Lentiviral AXL and p53 shRNA Constructs

Lentivirus preparations were produced by cotransfecting pLKO.1puro with *AXL* or *p53* shRNA and helper virus packaging plasmids Δ8.9 and VSVG into 293T cells. Transfections were carried out using Lipofectamine and PLUS reagent (Invitrogen Life Technologies, Carlsbad, CA, USA). Lentiviruses were harvested at 24, 36, 48, and 60 h post transfection. The virus was frozen at −80 °C in appropriately sized aliquots for infection. Well-validated shRNAs were used for AXL or p53 knockdowns, which had been evaluated in our previous published papers, including mesothelioma, ovarian cancer, and liposarcoma [11,28,31,44].

### 4.6. Cell Culture and Virus Infection

Mesothelioma cell lines (MESO924, MESO296, MESO428, and JMN1B) were maintained in RPMI 1640 with 10% fetal bovine serum (FBS) supplemented with penicillin/streptomycin and 1% (*v*/*v*) L-glutamine. The cells were seeded in six-well plates and lentiviral shRNA infections were carried out in the presence of 8 μg/mL polybrene. All lentiviral experimental results were performed in duplicate. Cells were lysed for Western blotting analysis at 96 h after infection. At least three independent assays were performed.

### 4.7. Transfection

Transfection of *AXL* or pCMV6 empty vector in HEK293T cells, and cotransfection of *AXL* and *TP53* expression vectors in COS-7 cells was carried out according to Invitrogen’s protocol, using lipofectamine and PLUS reagent. Briefly, DNA was incubated with PLUS reagent in 100 μL of serum-free medium (SFM) for 15 min at room temperature (RT). Lipofectamine was diluted with 100 μL of SFM and added to the DNA and PLUS complexes, and then incubated another 15 min at RT. Finally, DNA-PLUS-lipofectamine complexes were added to 60–70% confluent cultures of COS-7 or HEK293T cells in SFM in 800 μL of six-well plates, and incubated for 3 h, then were completely replaced with serum-containing regular media. Cells were lysed for Western blot analysis at 48 h post-transfection. At least two independent experiments were performed.

### 4.8. RNA Preparation and qRT-PCR

*AXL* and *TP53* RNA expression was evaluated by qRT-PCR after infection with *AXL* shRNA for 72 h in MESO924, MESO296, MESO428, and JMN1B. RT-PCR was performed using 1 μg prepared RNA by Trizol, with the iScriptTM cDNA synthesis Kit. qPCR was performed with iQ SYBR green supermix in a reaction volume of 25 μL, using a MyiQ single-color real-time PCR detection system (Bio-Rad). Reactions contained 1 μL cDNA, 400 nM each primer, and 12.5 μL iQSYBR green supermix. After 10 min at 95 °C, each of the 40 PCR cycles consisted of denaturation for 10 s at 95 °C and hybridization of primers and SYBR green as well as DNA synthesis for 1 min at 60 °C. The qRT-PCR assays for *AXL* and *TP53* were performed using the following primers: *AXL* (NM_001699): sense: 5′-GACCGGCCAAGTTTTACAGA -3′ and antisense: 5′-ATAACCTCCACCCTCATCCA-3′ [45]. *TP53* (NM_000546) sense: 5′-TAACAGTTCCTGCATGGGCGGC-3′ and antisense: 5′-AGGACAGGCACAAACACGCACC-3′ [46]. As a control, *GAPDH* (NM_002046) was amplified using the following primers: sense: 5′-GAAGGTGAAGGTCGGAGTCAAC-3′ and antisense: 5′-TGGAAGATGGTGATGGGATTTC-3′. The comparative Ct (cycle threshold) method was used to determine *AXL* and *TP53* expression differences in mesothelioma cell lines as compared to the MESO257 cell control. Data (run in triplicate assays) were normalized to *GAPDH*. Experiments were performed in triplicate.

### 4.9. Immunofluorescence Staining

MESO257 and MESO428 cells were seeded on laser confocal dishes (Solarbio, Beijing, China), then immobilized with 4% formaldehyde at RT for 15 min. The cells were washed three times with PBS (5 min/each time) and then permeabilized using 100% methanol at −20 °C for 10 min, and blocked using 5% bovine serum albumin (BSA) (Sigma, St Louis, MO, USA) and 0.5% Triton X-100 in PBS for 1 h at RT. Next, cells were incubated with primary antibodies for AXL (sc-166269, Santa Cruz Biotechnology. CA, USA) and p53 (#2527, Cell Signaling Technology, Shanghai, China) in 5% BSA overnight at 4 °C, and then rinsed in PBS (3 × 5 min, RT). The cells were then incubated with Alexa fluor 488 or 595 secondary antibody (Invitrogen Molecular Probes, Carlsbad, CA, USA) in 5% BSA in the dark for 1 h, at RT, and then rinsed in PBS (3 × 5 min, RT). Next, the cells were stained with DAPI (Beyotime, Shanghai, China) in the dark for 5 min at RT. Finally, immunofluorescence of the cells was visualized using a laser confocal microscope (TCS SPE ll, Leica, Germany). Experiments were performed in triplicate.

### 4.10. Dual Luciferase Assay

Dual luciferase assays were performed as published previously with minor modifications [28,37]. *TP53* firefly luciferase reporter construct *luc-p53* (0.25 μg), Renilla luciferase reporter *pTK-RL* (0.005 μg), and *AXL* construct or empty vector pCMV6 (0.25 μg) were cotransfected into 293T cells by using lipofectamine and PLUS reagent. Transfection of the pCMV6 empty vector alone served as a negative control in the reporter assays. The relative luciferase activity was calculated based on the amount of luminescence produced by Renilla luciferase for each reaction. Transfected cells were harvested after transfection for 48 h, and the reporter assay performed using a Dual-Luciferase Reporter Assay System, following the Promega′s instructions. Transfection efficiencies were normalized to the *pTK-RL* luciferase reporter. p53 luciferase activities were normalized with the pCMV6 vector. At least three independent experiments were carried out.

### 4.11. Chromatin Immunoprecipitation Assay (ChIP)

In total, 5 × 10^7^ MESO924 cells were prepared for ChIP assay according to the Simple ChIP^®^ Plus Enzymatic Chromatin IP Kit (Cell Signaling Technology, MA, USA). Briefly, the DNA of the crosslinked lysed cells was sheared by sonication for three times (each 10 s, and 30 s on the ice in intervals) and enzyme-digested to 200–500 bp length. Protein–DNA complexes were immunoprecipitated by AXL primary antibody (Santa Cruz, CA, USA) or control IgG antibody. The DNA fragment enriched by anti-AXL antibody was eluted and detected by RT-Q-PCR after dissociation of the protein–Ig–DNA complexes, The PCR primers are listed in Table S1.

### 4.12. Cell Viability Analysis

MESO924 cells were plated at 3000 cells per well in a 96-well flat-bottomed plate (Greiner, Frickenhausen, Germany) and cultured in RPMI 1640 for 24 h before infection with lentiviral pLKO or *p53 shRNA*, then 48 h later treated with AXL inhibitor R428 or infection with lentiviral *AXL shRNA*. Proliferation studies were carried out after 3 days using the MTT assay, and quantified using a Microplate Reader (TECAN, Grödig, Austria). The data were normalized to the empty vector group or DMSO. All the assays were performed in triplicate wells, and at least three independent experiments were performed for each cell line.

### 4.13. In Vitro Wound Healing Assays

MESO924 cell was plated in a 6-well flat-bottomed plate (Greiner, Frickenhausen, Germany) and cultured in RPMI 1640 for 24 h before infection with lentiviral pLKO or *p53 shRNA*. A slash was created using the 1 mL pipette tips at 48 h. The cell was immediately treated with AXL inhibitor R428 or infection with lentiviral *AXL shRNA*. The plates were photographed at 0 and 24 h using Spot software and a Nikon microscope. Experiments were performed in triplicate.

### 4.14. Cell Invasion Assays

For cell invasion assays, transwell chambers consisting of transwell-precoated matrigel and membrane filter inserts with 8 μm pores were used in 24-well cell culture plates (Costar, Cambridge, MA, USA). Cells from different groups (1 × 10^4^ per insert) were plated onto the top of the chamber in RMPI1640 without FBS and the bottom chamber was fed with RMPI1640 containing 10% FBS as a chemoattractant, meanwhile AXL inhibitor R428 was added to the upper and lower chambers to maintain a concentration of 1 μM. After 48 h of incubation in a 5% CO_2_ humidified chamber at 37 °C, the filter membrane was fixed with methanol and stained with 0.1% crystal violet, and non-invasion cells were removed by wiping the upper surface of the membrane with a cotton swab. The membrane on the chamber was cut into a 96-well plate and decolorized with a 33% acetic acid solution to read the OD value at 570 nm. The degree of invasion was quantified by counting the cells that had invaded through the membrane in at least four random fields (total magnification ×200) per filter. Experiments were performed in triplicate.

### 4.15. Statistical Analysis

Student’s *t*-tests were performed on data from cells treated with inhibitors/shRNAs or DMSO/pLKO (control). Statistically significant differences between control and treatment were defined as * *p* < 0.05, ** *p* < 0.01, and *** *p* < 0.001.

## 5. Conclusions

AXL negatively regulates p53 expression by binding to the first 600 bp at the 5′ end of the *TP53* promoter and inhibiting *TP53* transcription. AXL mediates tumor cell growth in mesothelioma through AXL/p53 signaling pathways. Targeting the AXL/p53 signal axis could be a useful treatment strategy in mesothelioma.

## Supplementary Materials

The following are available online at https://www.mdpi.com/2072-6694/12/10/2757/s1, Figure S1. *AXL* overexpression and prognosis in mesothelioma patients; Figure S2. Immunoblotting evaluation of the expression of AXL and p53 in mesothelioma total cell lysates; Figure S3. The whole blot (uncropped blots) showing; Table S1. Primers of chromatin immunoprecipitation-PCR (ChiP-PCR).
