# Correction: Song et al. AXL Inactivation Inhibits Mesothelioma Growth and Migration via Regulation of p53 Expression. *Cancers* 2020, *12*, 2757

**DOI:** 10.3390/cancers18162663

**Published:** 2026-08-18

**Authors:** Wei Song, Hao Wang, Minmin Lu, Xinxin Ni, Nacef Bahri, Shuihao Zhu, Limin Chen, Yuehong Wu, Jieqiong Qiu, Jonathan A. Fletcher, Wen-Bin Ou

**Affiliations:** 1 Zhejiang Provincial Key Laboratory of Silkworm Bioreactor and Biomedicine, College of Life Sciences and Medicine, Zhejiang Sci-Tech University, Hangzhou 310018, China; song786722358@163.com (W.S.); wanghao9611@126.com (H.W.); 15067150649@163.com (M.L.); nixin3877@163.com (X.N.); zsh19950701@163.com (S.Z.); clm2285183092@163.com (L.C.); wuyh77@zstu.edu.cn (Y.W.); qiujieqiong@zstu.edu.cn (J.Q.); 2 Department of Pathology, Brigham and Women’s Hospital and Harvard Medical School, Boston, MA 02115, USA; nbahri@bu.edu (N.B.); jfletcher@bwh.harvard.edu (J.A.F.)

## Figure Legend

In the original publication [1], there was a mistake in the legend for Figure 4. The source of Figure 4A was not appropriately attributed. The correct legend appears below. The authors state that the scientific conclusions are unaffected. This correction was approved by the Academic Editor. The original publication has also been updated.

AXL colocalization with p53 in the nucleus. (**A**) Reproduced from Fletcher et al. [24], licensed under CC BY-NC 4.0. Nuclear localization of AXL and p53 was evaluated in MESO257 by immunoblotting. Poly (ADP-ribose) polymerase (PARP) is a nuclear localization control, and GAPDH is a cytoplasmic control. The whole blot (uncropped blots) show in the Figure S3. (**B**) Cell distribution of AXL and p53 in MESO257 and MESO428 by immunofluorescence staining. The green stain represents AXL expression and red stain represents p53 expression. The color of image mergence of AXL (green) and p53 (red) stains showed orange. All the assays were performed from triplicate experiments.
